# Supplementary material for: MetaRibo-Seq measures translation in microbiomes
Source: Nat Commun. 2020 Jun 29;11:3268. doi: 10.1038/s41467-020-17081-z (PMC7324362; doi:10.1038/s41467-020-17081-z)
Supplement: Supplementary file 10 — Supplementary Data 7 [file 41467_2020_17081_MOESM10_ESM.zip › File2/Confidence_High_Taxonomy/306132_out.krona.html]

Javascript must be enabled to view this page.

members
magnitude
magnitudeUnassigned
count
unassigned
taxon
rank

306132\_out

18

18
superkingdom
2

18
1239
phylum

186801
class
18

18
order
186802

541000
family
18

18
1263
genus

species

SRS012273\_contig\_number\_43413SRS014235\_contig\_number\_47155SRS015854\_contig\_number\_8842SRS015890\_contig\_number\_contig-100\_193.163363SRS017916\_contig\_number\_17468SRS018984\_contig\_number\_33254SRS019601\_contig\_number\_16183SRS019685\_contig\_number\_40042SRS020233\_contig\_number\_contig-100\_759.343106SRS024492\_contig\_number\_contig-100\_118.114747SRS048060\_contig\_number\_contig-100\_674.84236SRS050752\_contig\_number\_25717SRS052697\_contig\_number\_contig-100\_276.259106SRS074964\_contig\_number\_contig-100\_1039.58683SRS098571\_contig\_number\_42736SRS142542\_contig\_number\_contig-100\_41.165683SRS147766\_contig\_number\_385SRS971275\_contig\_number\_172
46228
18
